# Supplementary figures and images for: Examining heterogeneity in dementia using data-driven unsupervised clustering of cognitive profiles
Source: PLoS One. 2024 Nov 14;19(11):e0313425. doi: 10.1371/journal.pone.0313425 (PMC11563363; doi:10.1371/journal.pone.0313425)

$K = 4$ 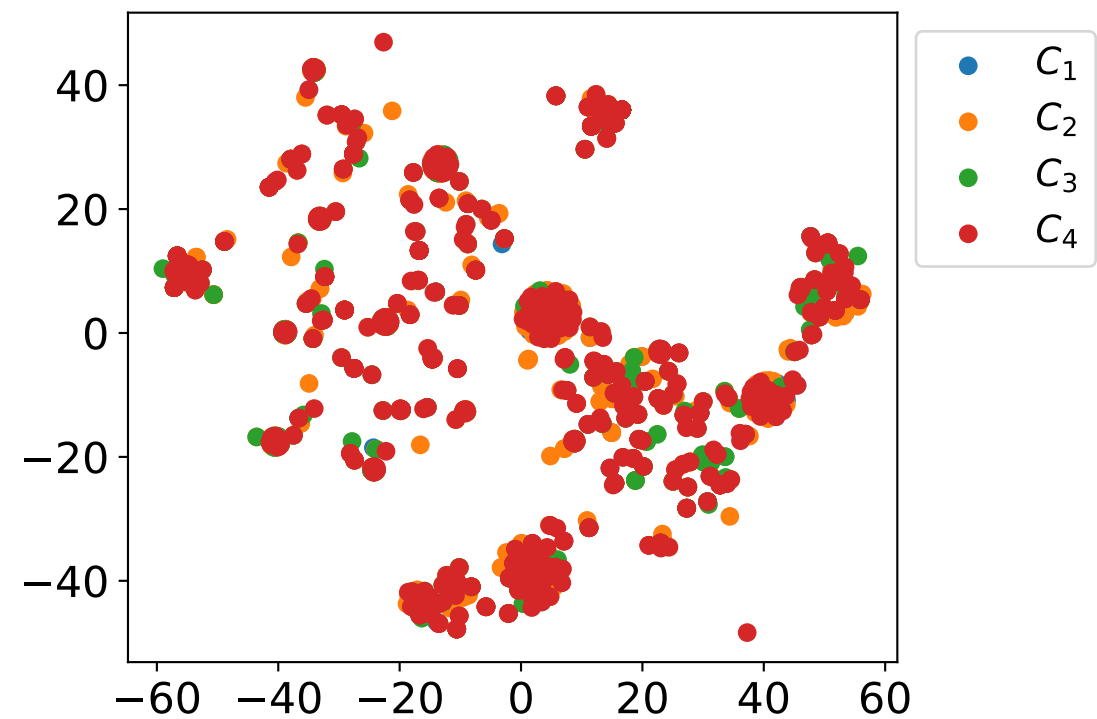 $K = 6$ 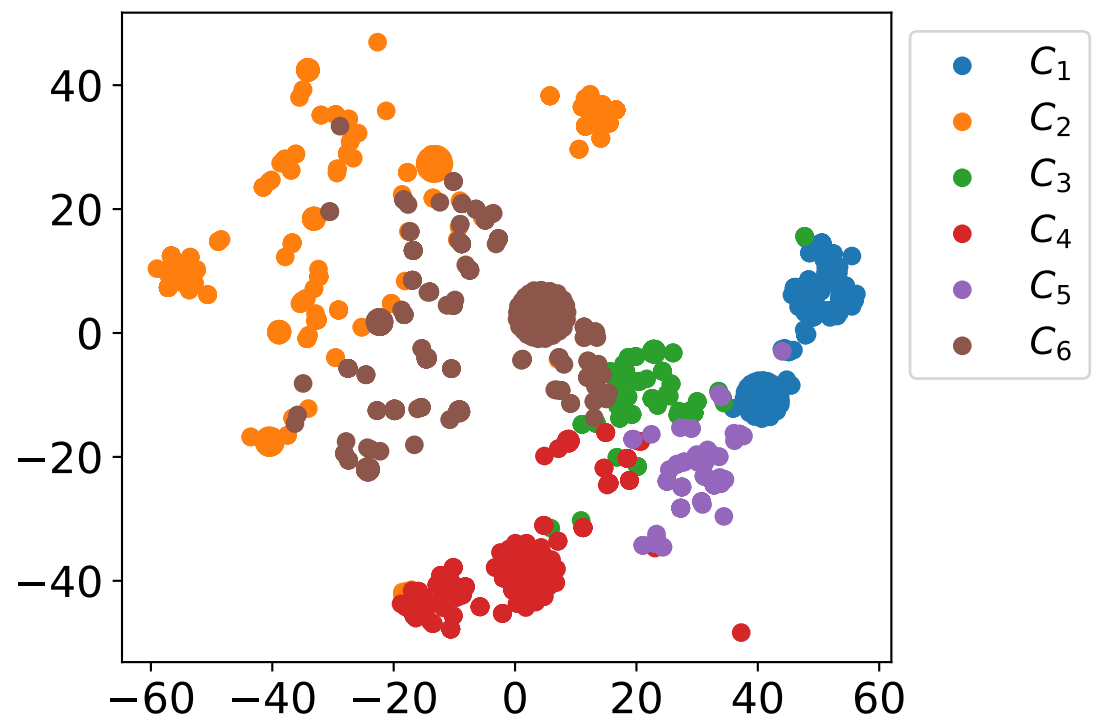 $K = 10$ 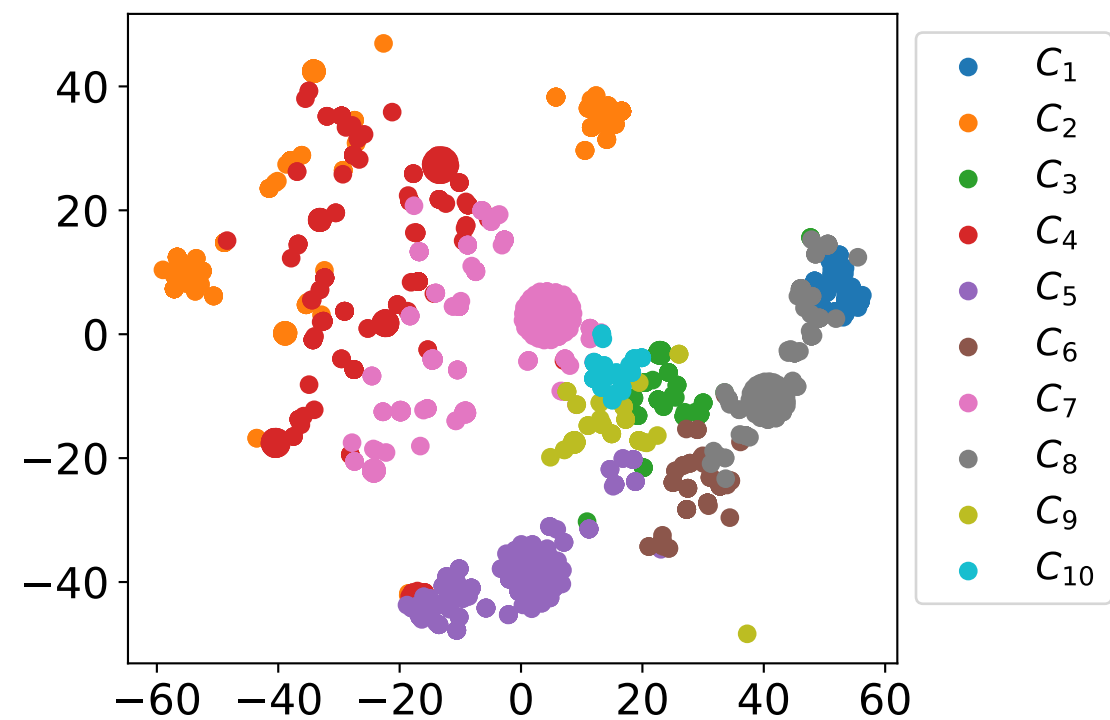 $K = 15$ 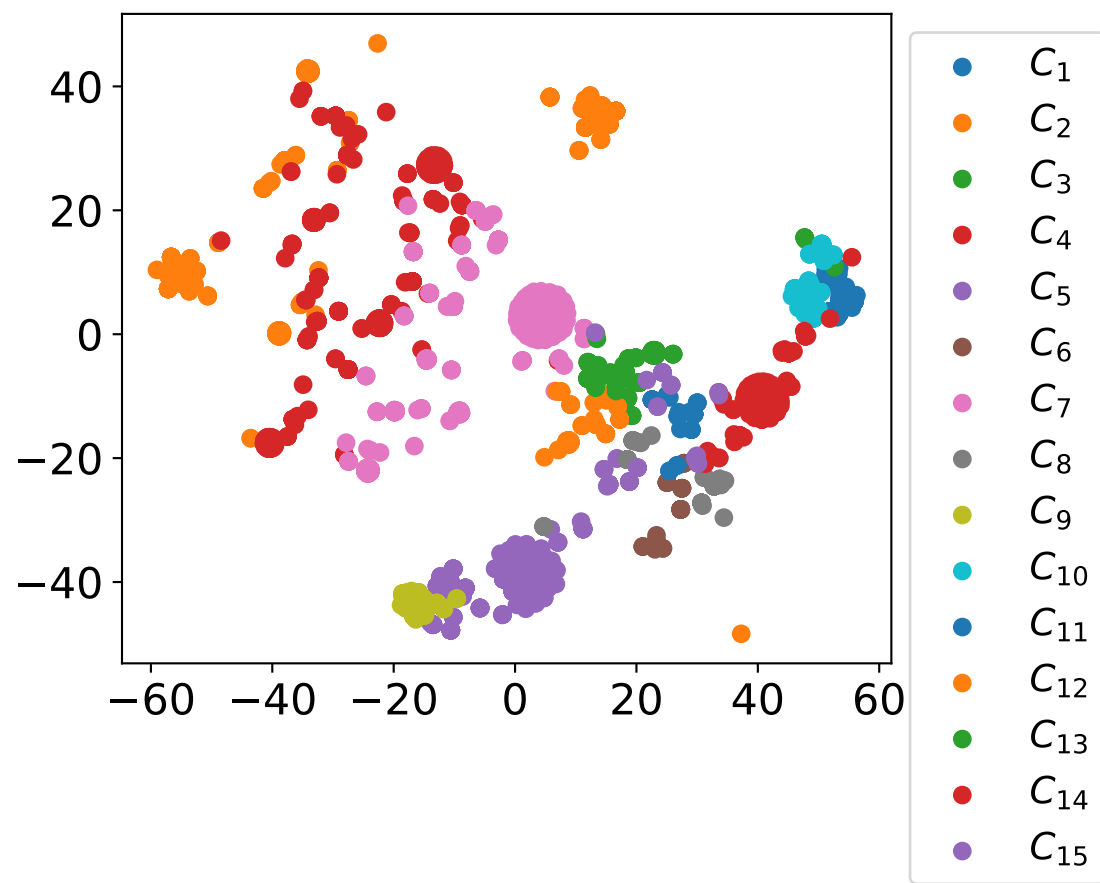

Supplement: S1 Fig — T-SNE distribution of the clustering results for different values of K = 4, 6, 10 and 15 respectively. The colorbar represents the different clusters. Each point in the scatter plot represents a single visit. The x-axis and y-axis represent the 2 dimensions of the 2D T-SNE vector for visualization purposes. (PDF) [file pone.0313425.s001.pdf]
