# Supplementary material for: Examining heterogeneity in dementia using data-driven unsupervised clustering of cognitive profiles
Source: PLoS One. 2024 Nov 14;19(11):e0313425. doi: 10.1371/journal.pone.0313425 (PMC11563363; doi:10.1371/journal.pone.0313425)
Supplement: S3 Table — The subtype groups in the columns and rows represent source and target respectively. The last column represents the total number of transitions from each source subtype. The diagonal values (marked in bold red) represent the number of self-transitions or instances where patients transition between the same subtype. Similar to Fig 5, the clusters are arranged in the order of increasing dementia severity from left to right. (DOCX) [file pone.0313425.s004.docx]

|  |  | **Source** | | | | | | | | | | |
| --- | --- | --- | --- | --- | --- | --- | --- | --- | --- | --- | --- | --- |
|  | **Subtype** | **C_2_** | **C_9_** | **C_4_** | **C_7_** | **C_5_** | **C_8_** | **C_6_** | **C_10_** | **C_3_** | **C_1_** | Total |
| **Target** | **C_2_** | **38** | 14 | 3 | 0 | 0 | 0 | 0 | 0 | 1 | 0 | **56** |
|  | **C_9_** | 15 | **75** | 24 | 13 | 7 | 1 | 0 | 1 | 0 | 0 | **136** |
|  | **C_4_** | 2 | 19 | **67** | 56 | 44 | 3 | 5 | 0 | 3 | 0 | **199** |
|  | **C_7_** | 0 | 1 | 7 | **54** | 50 | 15 | 5 | 9 | 8 | 9 | **158** |
|  | **C_5_** | 0 | 2 | 5 | 16 | **75** | 5 | 4 | 8 | 29 | 12 | **156** |
|  | **C_8_** | 0 | 0 | 0 | 8 | 4 | **10** | 2 | 2 | 1 | 0 | **27** |
|  | **C_6_** | 0 | 0 | 0 | 0 | 1 | 3 | **13** | 8 | 4 | 5 | **34** |
|  | **C_10_** | 0 | 0 | 0 | 1 | 2 | 1 | 6 | **12** | 8 | 7 | **37** |
|  | **C_3_** | 0 | 0 | 0 | 0 | 0 | 0 | 1 | 9 | **37** | 19 | **66** |
|  | **C_1_** | 0 | 0 | 0 | 0 | 0 | 0 | 0 | 1 | 3 | **19** | **23** |
