# Supplementary material for: Examining heterogeneity in dementia using data-driven unsupervised clustering of cognitive profiles
Source: PLoS One. 2024 Nov 14;19(11):e0313425. doi: 10.1371/journal.pone.0313425 (PMC11563363; doi:10.1371/journal.pone.0313425)
Supplement: Attachment — Submitted filename: reviewer_response_final.docx [file pone.0313425.s006.docx]

Dear editor and reviewers,

Thank you for the opportunity to revise and resubmit our manuscript (PONE-D-24-28122) entitled “*Examining heterogeneity in dementia using data-driven unsupervised clustering of cognitive profiles*”, for potential publication in PLOS One.

We appreciate the thoughtful and constructive comments by the reviewers and strongly believe it has considerably improved the manuscript.

We would like to thank the reviewers for their time in reviewing the manuscript and for their valuable feedback and suggestions. Below, we have addressed all the reviewer and editor comments in a point-by-point manner, which we hope would improve the quality of the manuscript and provide further clarifications. **All modifications in the revised manuscript have been highlighted in bold red.**

# **Comments from Editor**

## **Comment 1**

**Section 2.4**: how valuable do you think those data are from patients with single visits? Do you not think that single visits may just be anecdotal evidence, and thus have less statistical power? According to section 3.1, this covers 953 patients, more than half of all patients. Would the analysis be different if you excluded those patients with one visit only? With only one visit you cannot measure transition between stages (section 3.6).

## **Response**

Thank you for your thoughtful comments. We agree that patients with a single visit cannot contribute to the analysis of transitions between stages. As you correctly noted, to measure transitions, we included only patients with multiple visits and excluded all patients with a single visit. **We have clarified this point in section 3.6 (“*cluster transitions*”) of the revised manuscript**.

However, we included all patients, including those with a single visit, in the clustering analysis to maximize the sample size and enhance the statistical power of the clustering algorithm. Including these patients helped to more accurately define cluster characteristics and ensured that our model captured the full spectrum of the cohort's heterogeneity. While patients with a single visit do not contribute to longitudinal transitions, their inclusion in the clustering process allowed us to generate more robust and representative clusters, which were then applied to the longitudinal cohort for transition analysis. **We have clarified the above point in the Discussion section (end of 5^th^ paragraph) of the revised manuscript.**

## **Comment 2**

**Section 3.1**: please describe what ICD is and what these codes mean in the text for those who do not know what they mean

## **Response**

Thank you for this comment. ICD (International Classification of Diseases) codes are a standardized set of alphanumeric codes used globally to classify diseases, symptoms, medical conditions, and procedures. They are maintained by the World Health Organization (WHO) and are used in healthcare settings for documentation, billing, and research purposes. Each ICD code represents a specific diagnosis or health-related issue, allowing consistent communication across healthcare systems and researchers. **The full list of ICD codes for each of the brain-related disorders listed in Table 1 can be found in the Supporting Information (S2 Table).** **A brief definition of ICD has also been added in Section 3.1.**

## **Comment 3**

**Section 3.3.1**: why did you select k=10 optimal clusters? According to the scree plot the elbow portion seems to be at k=5.

## **Response**

Thanks you for this comment. Here is a detailed explanation/justification of our rationale behind selecting K=10 as the optimal number of clusters.

Comparing the mean silhouette width (MSW) values for different number of clusters we identified K = 4, 6, 10, 15 as potential candidates where we observed increase in MSW compared to their neighbouring K value points (Fig 3). This aligns with T-SNE distributions (Fig 2B, Supporting Information S3 Fig), showing that higher number of clusters (K = 10, 15) better capture heterogeneity in cognitive profiles amongst early dementia patients (CDR = 0.5) compared to lower values of K (K = 4 6). Choosing a lower value of K merges several smaller subclusters, which limits the ability to explore heterogeneity in greater detail. As K = 10 and K = 15 produced similar results, with K = 15 only further subdividing clusters for CDR > 1, we selected K = 10 as the optimal number of clusters, as our primary focus was to examine heterogeneity in the early stages of dementia (CDR ≤ 1). **We have highlighted these justifications in section 3.3.1 (“*optimal number of clusters*”) of the revised manuscript.**

## **Comment 4**

**Section 3.4**: how can we tell from figure 4 which each cluster (C1–10) belongs to (mild, severe, etc.)?

## **Response**

Thank you for this comment. The purpose of Figure 4 is to show how each clustering method (Hierarchical SillyPutty and the baselines) identified the clusters. Hence the numbering and arrangement of the clusters is somewhat arbitrary. The cluster numbers in Figure 4 (C1-C10) represent the 10 clusters in numerical order. The same clusters are arranged in order of increasing dementia severity in Figure 5 where we can observe the CDR composition of each of the clusters.

## **Comment 5**

**In figure 5** clusters C5 and C7 look like they have similar composition of CDR 0.5 and 1, why not unite these 2 clusters? Same thing for C6 and C10.

## **Response**

Thank you for this comment. While clusters C5 and C7 do look similar in their CDR composition of CDR 0.5 and 1 (Figure 5), we would like to point out that they have different distribution of CDR component scores, particularly “judgement & problem solving”, “community affairs”, “home & hobbies” and “personal care” (Figure 6). C5 also has a higher risk of progression, demonstrated by a higher number of patient transitions to CDR > 1 (Figure 7). Similarly, C6 and C10 even with similar CDR composition of CDR 1 and 2 (Figure 5) have variability in the distribution of memory, orientation, community affairs and personal care scores (Figure 6). Our main goal in this work was to investigate the extent of heterogeneity amongst patients at similar level of dementia severity (same CDR score). Choosing a higher number of clusters (K = 10 instead of K = 4 or 6; Figure 3) enabled a more detailed examination of the heterogeneity in cognitive profiles and risk of disease progression.

# *******************************************************************************************

# **Comments from Reviewer: 1**

This paper uses a recent clustering algorithm (SillyPutty) to process and analyze dementia in a data set that includes many patients and several years of information. The authors deal with limitations that characterize previously published work and successfully provide results while considering the heterogeneity of the patients and their symptoms. Exploiting easily obtained measurements and features allows for a clear definition of dementia subtypes, as the adopted and enhanced data clustering approach specifies. Moreover, with their data organization and post-processing procedure, the authors explore and manage to connect the subtypes with the progression of dementia over time.

It is not easy to find flaws in this interesting and high-quality work. The text is very well organized and easy to read. The work is technically sound and uses state-of-the-art methods and data of great scientific interest. The experimental results and the conclusions drawn are very clear. This work advances the current state-of-the-art and provides a strong motivation for applying the presented methodology at a significant larger scale.

## **Response**

Thank you for your thorough review and positive comments on the manuscript. We have responded in a point-by-point manner to your minor comments below.

## **Minor Comment 1**

Some typos must be corrected in the final version of the paper (e.g. patents instead of patients)

## **Response**

Thank you for pointing this out. **We have made sure to correct all typos in the revised version of the manuscript.**

## **Minor Comment 2**

The naming of the clusters (C1-C10) is based on the results of the clustering method. It might be beneficial if these names are mapped to a new set of names (e.g. D1-D10), where the index is following the dementia severity (i.e. C2->D1, C9->D2).

## **Response**

Thank you for the suggestion. **While we acknowledge the potential benefits of renaming clusters to follow dementia severity (e.g. D_1_-D_10_), we opted to retain the current naming convention (C_1_-C_10_) for clarity and consistency with the clustering results.** Particularly, since some clusters share the same dementia severity level (e.g., C9 and C4 both have CDR = 0.5), using severity-based naming convention would result in duplicate names for clusters with identical CDR levels.

## **Minor Comment 3**

CRAN also includes density-based clustering algorithms (e.g. DBSCAN) that do not require the user to specify the number of clusters. They might not be suitable for the specific dataset, but some reference to those algorithms, at least, would be useful.

## **Response**

Thank you for highlighting density-based clustering algorithms like DBSCAN. While we acknowledge that such methods do not require the number of clusters to be pre-specified and can be useful for detecting irregularly shaped clusters, preliminary testing indicated that these approaches were less effective for our specific dataset. The nature of our data, which includes highly structured clinical profiles (CDR components), made other clustering methods more appropriate for this analysis.

For our work, we used hierarchical SillyPutty, which is a state-of-the-art clustering technique and has been demonstrated to have better clustering performance than commonly used clustering techniques[1]. **As suggested by the reviewer, we have included a reference to DBSCAN in the Methods (section 2.3.3; “*SillyPutty with hierarchical clustering*”) of the revised manuscript. Following the recommendation of Reviewer 3 (comment 3), we have also compared the mean silhouette width (MSW) of SillyPutty and other commonly used clustering techniques K-means and DBSCAN in the Results (section 3.3.2; “*comparison with other clustering methods*”) in the revised manuscript.**

[1] Bombina P, Tally D, Abrams ZB, Coombes KR. SillyPutty: Improved clustering by optimizing the silhouette width. Plos one. 2024 Jun 7;19(6):e0300358.

## **Minor Comment 4**

The results (percentages) in Table 2 (Cluster demographics) probably need clarification: For example, the percentage of White Race in Table 1 is 77%, but the overall corresponding percentage in Table 2 is much higher (its lowest value is 83.3% for variable C8). Is this due to the "baseline visits" of Table 1 and the "all visits" for Table 2?

## **Response**

Thank you for your observation. The difference in percentages between Table 1 and Table 2 is indeed due to the fact that Table 1 reflects demographic data based on baseline visits, while Table 2 represents demographic data across all visits. Since Table 2 accounts for multiple visits per individual, certain demographics, such as race, may appear more frequently due to the longitudinal nature of the dataset. **To maintain consistency between the two tables, we have updated Table 2 to reflect baseline visits only, ensuring a more accurate comparison with the demographic percentages in Table 1. We have also clarified this in the caption of Table 2 and section 3.4 (“*subtype demographics*”) in the revised manuscript.**

# *******************************************************************************************

# **Comments from Reviewer: 2**

The authors argue that the growth of Electronic Health Records has opened new data-driven approaches to the heterogeneous trajectories associated with dementia. In their view, existing approaches have been limited by an emphasis on expensive neuroimaging and single time-point assessment. They, conversely, investigate the possibilities of trajectories in more widespread cognitive assessment tasks. Using the health records of visitors to a university memory clinic over a number of years, they first use unsupervised learning to identify different clusters of scores on a multidimensional assessment instrument and then recreate trajectories in terms of transitional probabilities between these clusters.

I think the authors make a good case for the value of this project. If useful information on dementia trajectories can be extracted from widely used tests, it would provide an efficient way to ground better assessments of risk and (potentially) personalization of treatments.

I also applaud the authors' clear and concise explanation of their approach and procedures. The paper was to the point, focused, and enjoyable to read.

In terms of minor points, there were a couple of places where things could be explained a little further.

## **Response**

Thank you for your thorough review and positive comments on the manuscript. We have responded in a point-by-point manner to all your comments below.

## **Minor Comment 1**

The description of measures on page 5 is not easy to understand; I was unclear if the history / neurological examination, the cognitive assessment battery and the CDR were three separate things or not. The list on page 6 makes it much clearer, so I'd suggest moving some of this information up to the earlier section.

## **Response**

Thank you for your feedback on the clarity of the measures described. We agree that the distinction between the history/neurological examination, cognitive assessment battery, and Clinical Dementia Rating (CDR) could be clearer in the earlier section. **To address this, we have revised the description on page 5 (section 2.1; “*data sources and study participants*”) to better differentiate these components. Further, we have added information from the list of cognitive assessment scores and CDR components on page 6 (section 2.2.1; “*feature selection for clustering*”) to improve clarity.** This should help readers understand the structure and distinctiveness of the different terminologies from the outset.

## **Minor Comment 2**

Similarly, describing the dataset in the materials section without giving sample sizes (which came in the Results section) threw me a bit. I think it would be more comprehensible if the size of the data is mentioned earlier.

## **Response**

Thank you for pointing out the need for better placement of the sample size information. We agree that introducing the sample sizes earlier in the Methods (section 2.1; “*data sources and study participants*”) would enhance the comprehensibility of the dataset description. **We have added the sample size information in section 2.1 to provide a clearer context for readers as they proceed through the manuscript.**

## **Minor Comment 3**

The data period on page 5 seems to be five years, but on page 8 is described as six years.

## **Response**

We appreciate your attention to this detail. The discrepancy in the data period was unintentional, and we will correct it to ensure consistency. The accurate data period is five years. **We have updated both instances in the manuscript (section 2.1; “*data sources and study participants*” and section 2.4; “*clustering on longitudinal visits*”) to reflect the correct time period of the data.**

## **Minor Comment 4**

The rationales for clustering on CDR components (page 10) and for choosing 10 as the optimal number of clusters (page 11) could be filled out a bit more. Right now a few considerations are given, but I didn't quite understand what the deciding factor was in these decisions.

## **Response**

Thank you for your feedback. Here is a brief explanation/justification of the rationales for selecting CDR components as the optimal features for clustering and choosing K=10 as the optimal number of clusters.

**CDR components as the optimal feature set for clustering**:

The choice behind selecting CDR components as the optimal set of features for clustering can be justified in a both qualitative and quantitative way.

**Qualitative**: We qualitatively analyzed the T-SNE distribution of two feature sets (CDR components and cognitive assessment scores) to determine the optimal input features for clustering. Cognitive scores created a gradient aligned with global CDR but did not produce distinct clusters (Fig 2A). In contrast, the CDR components produced well-separated clusters without significant overlap (Fig 2B), making them the most suitable features for clustering.

**Quantitative:** Following the recommendation of Reviewer 3 (comment 1) , we conducted PCA on both the cognitive assessment scores and the CDR components. Our results show that the first 2 principal components of the CDR components explain a significantly larger proportion of the variance (**81.6 %** and **12.7%** respectively) compared to the cognitive assessment scores (**75.8%** and **14.5%** respectively). This quantitatively supports our decision to use CDR components as the primary features for clustering. **We have added both the justifications in section 3.2 (“*optimal set of features for clustering*”) of the revised manuscript.**

**Optimal number of clusters (K = 10)**:

Comparing the mean silhouette width (MSW) values for different number of clusters we identified K = 4, 6, 10, 15 as potential candidates where we observed increase in MSW compared to their neighbouring K value points (Fig 3). This aligns with T-SNE distributions (Fig 2B, Supporting Information S3 Fig), showing that higher number of clusters (K = 10, 15) better capture heterogeneity in cognitive profiles amongst early dementia patients (CDR = 0.5) compared to lower values of K (K = 4 6). Choosing a lower value of K merges several smaller subclusters, which limits the ability to explore heterogeneity in greater detail. As K = 10 and K = 15 produced similar results, with K = 15 only further subdividing clusters for CDR > 1, we selected K = 10 as the optimal number of clusters, as our primary focus was to examine heterogeneity in the early stages of dementia (CDR ≤ 1). **We have added these justifications in section 3.3.1 (“*optimal number of clusters*”) of the revised manuscript.**

## **Minor Comment 5**

The strategy for examining cluster transitions was under-described. First, I didn't see mention of positive transitions (i.e. from more severe to milder clusters) - an examination of Table S4 suggests these are quite rare, so I assume it was a deliberate analytic choice to focus on the more common case of increasingly severe impairments. Second, the analysis is currently at the level of raw descriptive statistics; it would be useful to translate these into transitional probabilities and perform inferential testing.

## **Response**

Thank you for your feedback on the cluster transitions the need for inferential testing on transitional probabilities. We address both the above points below.

**Reverse transitions (severe to mild dementia)**: As mentioned by the reviewer, we focused primarily on the more common case of forward transitions toward increasingly severe impairments (mild to severe dementia), as dementia is generally a progressive and irreversible condition. Reverse transitions (i.e., from more severe to milder clusters) on the other hand were rarely observed in our study (Supporting Information S4 Table). The regression in CDR score could be potentially related to treatment of conditions that can cause or worsen dementia symptoms. For example, dementia specialists often discontinue medications that impair cognition, and patients often improve after these changes are made. **We have briefly discussed the reverse transitions and their potential cause in the Discussion section (section 4, paragraph 4) of the revised manuscript.**

**Inferential testing on transitional probabilities:** While we agree that translating the transitions into probabilities and performing inferential testing could offer additional insights, the limited sample size makes it challenging to conduct robust inferential analysis. The main motivation behind our analysis was to analyze the initial patterns of heterogeneity seen within a dementia cohort and a possible solution of parsing the heterogeneity by characterizing the dementia subtypes. Due to the limited number of patients with subtype transitions corresponding to disease progression (N = 347), we opted for descriptive statistics to provide an initial overview of the transition patterns. **We have acknowledged this as a limitation in the Discussion section (section 4, paragraph 6) of our revised manuscript.** **Applying this pipeline to a larger EHR dataset can allow us to estimate probabilistic estimates of disease progression, backed by rigorous statistical analysis. We believe this can be an important scope for future work.**

## **Major Comment**

However, I feel the greatest weakness of the paper was in not making the case for its clustering approach (as compared to other modelling approaches). The authors make a comparison of different clustering algorithms, but not different types of model. There are a couple of salient alternative approaches, depending on the key research goal. If the aim is to better understand trajectories, latent growth curve methods or hierarchical linear models may be relevant. If the goal is to predict risk, supervised learning methods such as regularized regression may have value (predicting changes from visit to visit). The key value of a clustering approach over these approaches is its ability to model heterogeneity; rather than a single spectrum of severity, clustering can capture common profiles of scores across a number of dimensions. However, broadly, the resulting clusters look like a spectrum of increasing severity (see e.g. Table 3); there is some heterogeneity (e.g. transitions from mild to moderate CDR scores was more likely for those with more functional impairment), but this seems minor and reduces as the condition gets more severe. As such, I wonder what added value the clustering approach is providing in this particular case. Why not just model global CDR scores as the key outcome of interest, and then use CDR component and cognitive battery scores as additional predictors? Ultimately it seems like this is where the clustering analysis ends up.

## **Response**

Thank you for your insightful comments and suggestions. We agree that different modeling approaches, such as latent growth curve models or supervised learning methods, can be useful depending on the research goal. However, our decision to use clustering was deliberate and motivated by the need to model heterogeneity in cognitive and functional impairment profiles.

**Clustering advantages**: Unlike supervised methods that focus on predefined outcomes, clustering allows us to uncover naturally occurring subgroups within the data that may not align with linear trajectories of severity. The key value of clustering in our analysis is to capture these multidimensional profiles across cognitive and functional measures, which provides a more holistic view of patient variability. This approach is especially useful for understanding the distinct, co-occurring impairments beyond a single spectrum of severity.

**Heterogeneity in clusters:** While we acknowledge that some clusters may appear to reflect increasing severity (as seen in Table 3), our analysis also reveals meaningful heterogeneity, especially in patients with very mild to mild dementia (CDR = 0.5, 1). For example, clusters (subtypes) C4 and C9 in Figure 5 shows that subtypes can be in the same level of dementia severity (CDR = 0.5) but can show significant variability in their cognitive and functional impairments (Figure 6) and their risk of progression (Figure 7). Our results also indicated that subtypes with visits having mild dementia (CDR = 0.5/1) were more heterogenous in their cognitive profiles compared to subtypes with visits having moderate to severe dementia (CDR > 1) Identifying and diagnosing individuals in the initial stages of their cognitive decline early can enable timely intervention, which might slow the progression of the disease and improve the quality of life.

**Modeling global CDR score as the key outcome of interest:** Global CDR scores, while useful, offer a more one-dimensional view of disease progression and cognitive decline due to dementia. As demonstrated from Figure 6, Figure 7 and Table 3, patients with the same global CDR score can have considerable variability in cognitive, functional impairments and risk of disease progression. By clustering, we leverage the individual CDR components to capture complex profiles, which allows for a richer characterization of disease trajectories. We believe that this multidimensional view cannot be fully achieved through a simple linear progression of global CDR scores.

# ******************************************************************************

# **Comments from Reviewer: 3**

After thoroughly reviewing the manuscript, I would like to commend the authors for their innovative approach and the valuable insights provided into dementia heterogeneity. However, several key methodological and analytical aspects require significant enhancement to strengthen the validity and impact of the findings. The current approach, while promising, would benefit greatly from incorporating additional analyses and more rigorous justifications. I recommend a major revision to address these critical points and improve the overall robustness and clarity of the study.

## **Response**

Thank you for your thorough review and insightful comments on the manuscript. We have responded in a point-by-point manner to all your comments below.

## **Comment 1**

The authors chose to use the six CDR components as features for clustering rather than the cognitive assessment scores, based on observation that CDR components better differentiate the patient clusters. But t-SNE is primarily a tool for data visualization (reducing data dimensionality to visualize the structure in a way that preserves the local distances between points), it is not a traditional feature selection method and doesn’t provide a direct measure of the importance or variance explained by different features. I suggest the authors consider supplementing with PCA. If PCA on both cognitive scores and CDR components show that the first few principal components of the CDR data explain a significant proportion of the variance (more so than the cognitive scores), then this would quantitatively support using CDR components as the primary features for clustering. Or conversely, if (cognitive scores) or (cognitive scores + CDR components) explained more variance, then the authors might reconsider their features to include those that contribute most to distinguishing between patient subtypes.

## **Response**

We thank the reviewer for this valuable suggestion. We agree that using TSNE visualization is only a qualitative way of optimal feature selection and doesn’t provide a direct measure of the importance or variance explained by different features. Following this recommendation, we conducted PCA on both the cognitive assessment scores and the CDR components. Our results show that the first 2 principal components of the CDR components explain a significantly larger proportion of the variance (**81.6 %** and **12.7%** respectively) compared to the cognitive assessment scores (**75.8%** and **14.5%** respectively). This quantitatively supports our decision to use CDR components as the primary features for clustering. **We have also discussed this PCA experiment in section 2.2.1 (“*Feature Selection for clustering*”) and the PCA results in section 3.2 (“*Optimal set of features for clustering*”) respectively.**

## **Comment 2**

The decision to combine SillyPutty with hierarchical clustering is well-justified based on previous studies. But can the authors provide more details on the initialization parameters, the number of iterations, and how convergence was assessed etc.?

## **Response**

Thank you for this question. Details regarding the initialization parameters, number of iterations and convergence conditions are below. **We have also highlighted these details in the revised manuscript (section 2.3; “*Unsupervised clustering*”)**

**Initialization parameters**: As reported in the original SillyPutty paper [1], the standalone SillyPutty algorithm starts with purely random cluster assignments, repeating the algorithm with (by default) 100 different random starting points. However, SillyPutty can also start with any cluster assignment generated by conventional clustering techniques e.g., hierarchical clustering. For our work, we followed the recommendation of the original paper and used hierarchical SillyPutty (SillyPutty initialized with hierarchical clustering).

**Number of iterations**: Maximum number of iterations limit was set to 100.

**Convergence**: The SillyPutty algorithm continues until all data points have non-negative silhouette widths, or an early termination condition is reached. SillyPutty algorithm terminates under any of the 3 conditions:

- When all data points are correctly classified, indicated by all silhouette widths being non-negative, signalling convergence.
- When the maximum allowed iterations are reached (N = 100), to prevent the process from running indefinitely.
- When the algorithm identifies a potential infinite loop, detected by encountering the same silhouette width vector within the last n iterations, where n is a user-defined parameter.

[1] Bombina P, Tally D, Abrams ZB, Coombes KR. SillyPutty: Improved clustering by optimizing the silhouette width. Plos one. 2024 Jun 7;19(6):e0300358.

## **Comment 3**

The SillyPutty approach is innovative, but I think it’s important to understand how it compares with other established clustering methods more widely recognized in the ML community. E.g. k-means, which is a common baseline method in clustering tasks with presumably much smaller computational costs than SillyPutty, or DBSCAN which is suitable for irregularly shaped clusters and can handle noise or outliers well (relevant in clinical data where not all data points neatly fit into clusters). An informative addition would be to compare the average the silhouette width of SillyPutty against DBSCAN, k-means etc, so we know whether potentially simpler methods can achieve comparable results. If SillyPutty shows clear advantages over other methods in the context of dementia research, this could position it as a valuable tool for other researchers/clinicians. Conversely, if other methods perform similarly or better in some respects, then SillyPutty could be more useful in specific scenarios or with certain types of data.

## **Response**

We appreciate the reviewer’s suggestion to compare SillyPutty with other established clustering methods such as K-means and DBSCAN. We would like to point out that the original SillyPutty paper [1] evaluated the average silhouette widths of multiple state-of-the-art clustering techniques such as K-means, hierarchical clustering, PAM (partition against medoids), spectral clustering, CLARA (clustering large applications) etc. and found hierarchical SillyPutty to the best performing clustering method (maximum average silhouette width). **To further address this, we performed a similar comparative analysis between hierarchical SillyPutty and state-of-the-art clustering techniques including K-Means and DBSCAN.** Our results demonstrated that hierarchical SillyPutty showed superior performance compared to the other techniques had higher average silhouette width compared to popular techniques like K-means and DBSCAN. This results in the context of our study positions SillyPutty as a valuable tool in dementia research and other clinical applications. **The comparative analysis described above has been described in the Results section (section 3.3.2; “*comparison with other clustering methods*”)**

[1] Bombina P, Tally D, Abrams ZB, Coombes KR. SillyPutty: Improved clustering by optimizing the silhouette width. Plos one. 2024 Jun 7;19(6):e0300358.

## **Comment 4**

Relying solely on silhouette width might give an incomplete picture of the clustering. Including multiple metrics e.g. DBI, Dunn, would make the manuscript’s argument for the effectiveness of SillyPutty more convincing by showing rigorous evaluation from multiple angles, not just a single metric. SillyPutty is the first algorithm to use silhouette width as a quantity to be optimized directly when finding clusters. It is important to note that in this case silhouette width is optimized directly and not used as a comparative tool to find the optimal number of clusters or evaluate the cluster

## **Response**

We appreciate the reviewer’s suggestion to include additional metrics such as DBI and Dunn Index for evaluating the clustering performance. However, we would like to clarify that SillyPutty is distinct from traditional clustering algorithms, as it directly optimizes silhouette width during the clustering process, rather than using it solely as a comparative evaluation tool. This makes the algorithm unique, as silhouette width becomes the objective function guiding the clustering, rather than being a post-hoc measure of performance. While DBI and Dunn Index are useful metrics for evaluating clustering quality, they are less relevant in this context, as the primary goal of SillyPutty is to maximize silhouette width during clustering.

## **Comment 5**

Treating each visit as an independent data point in the clustering process is potentially problematic, because in a clinical context (especially with chronic and progressive conditions like dementia), the cognitive status of a patient at one visit is likely influenced by their status at previous visits. The authors should acknowledge the potential limitations of this assumption of independence, and discuss how this might affect the interpretation of their findings. Could also consider highlighting alternative approaches like HMM as avenues for future research, which would demonstrate a forward-thinking approach and acknowledge the complexities of analyzing longitudinal clinical data.

## **Response**

We thank the reviewer for this important point regarding the assumption of independence across visits. We would like to emphasize that our design choice of treating each visit as an independent data point in the clustering process was motivated by the following reasons. First, analyzing visit-level data allows us to capture temporal patterns and transitions between dementia subtypes, providing insights into disease progression that a baseline-only approach cannot offer. Second, treating each visit as a separate data point increases the data volume, which is crucial for enhancing statistical power, especially in smaller cohorts. Finally, our EHR data spans only six years (2012-2018) and includes patients with varying clinical windows, making it necessary to use all visits to conduct a more comprehensive longitudinal analysis.

However, we acknowledge the reviewer’s comment that cognitive status at one visit may be influenced by prior visits, particularly in progressive conditions like dementia. While our current approach focuses on clustering independent data points for the reasons mentioned above, we acknowledge this as a limitation in our analysis. **We have now included a statement in the Discussion section (limitations) of the revised main manuscript to address this point. Further, we have also highlighted in the Discussion section that future research could explore more sophisticated longitudinal approaches, such as Hidden Markov Models (HMM), to better capture the temporal dynamics of cognitive decline and progression in dementia.**

## **Comment 6**

The manuscript identifies heterogeneity in cognitive profiles, particularly in the early stages of dementia. I believe the discussion could be more nuanced in exploring e.g. why certain clusters exhibit different progression risks despite similar initial profiles. What about potential confounding factors that could influence these findings, such as comorbidities, medication effects or SES?

## **Response**

We appreciate the reviewer’s insightful suggestion regarding potential confounding factors such as comorbidities, medication effects, and socioeconomic status (SES). We acknowledge that our experiments did not consider the above potential confounding factors which could potentially influence the progression risks of certain clusters despite similar initial profiles. Incorporating additional data on comorbidities, medication, and SES to better understand their impact on cluster progression can be an important avenue for further research. **We have also added this point as a limitation in the Discussion section of our revised manuscript.**

## **Comment 7**

The study's findings have implications for personalized dementia care, particularly in identifying patients at higher risk of rapid progression. And the manuscript could better connect these findings to specific clinical interventions or decision-making processes, e.g. how could identifying "progressive MCI" subtypes influence treatment plans or monitoring strategies?

## **Response**

We appreciate the reviewer’s suggestion and agree that identifying "progressive MCI" subtypes could significantly influence clinical practice. By recognizing these subtypes early, clinicians can tailor treatment plans to the specific progression risk of each patient. For instance, patients identified as being at higher risk for rapid cognitive decline (e.g. C4) could benefit from more aggressive therapeutic interventions (e.g., early pharmacological treatment, lifestyle modifications) or more frequent cognitive monitoring. Additionally, this stratification could help prioritize patients for clinical trials targeting slowing cognitive decline, thereby optimizing resource allocation and improving patient outcomes. **This has been clarified and highlighted in the Discussion section of the manuscript.**

Sayantan Kumar, MS

Department of Computer Science and Engineering

Institute for Informatics, Data Science and Biostatistics (I2DB)

Washington University School of Medicine

[sayantan.kumar@wustl.edu](mailto:sayantan.kumar@wustl.edu)
